# Assessment of functional movement screen and performance parameters of wrestlers using inertial sensors

**DOI:** 10.3389/fspor.2025.1560924

**Published:** 2025-06-25

**Authors:** Batbayar Khuyagbaatar, Batlkham Dambadarjaa, Zul Altan-Ochir, Ganzorig Battumur, Erdenevaanchig Batbaatar, Yoon Hyuk Kim

**Affiliations:** ^1^ Biomechanical Research Laboratory, Department of Mechanical Engineering, Mongolian University of Science and Technology, Ulaanbaatar, Mongolia; ^2^ Department of Physical Therapy, Mongolian National University of Medical Sciences, Ulaanbaatar, Mongolia; ^3^ Department of Research, Sports Medicine and Research Center, Ulaanbaatar, Mongolia; ^4^ Department of Mechanical Engineering, Kyung Hee University, Yongin, Republic of Korea

**Keywords:** functional movement, performance, wrestling, kinematics, inertial sensors

## Abstract

A functional movement screen (FMS) is an assessment system that identifies athletes' movement profiles and injury risks. This is also used to determine sport-specific performance and training effectiveness. However, none of the studies have employed the IMU measurement system to assess FMS and performance parameters in wrestling. In this study, we aimed to assess FMS and kinematic parameters in wrestlers using IMU sensors to explore the relationship between FMS scores, range of motion (ROM), and performance parameters. Ten healthy controls and ten wrestlers completed the seven tasks of the FMS and performed wrestling techniques. The screening results were assessed, revealing significant differences in shoulder mobility (Control: 2.7 ± 0.6, Wrestlers: 1.9 ± 0.8) (*p* = 0.034, Cohen's *d* = 1.02) and active leg raise tests (Control: 2.3 ± 0.4, Wrestlers: 2.9 ± 0.3) (*p* = 0.004, Cohen's *d* = 1.47) between the two groups. Additionally, center of mass (CoM) velocity increased by 18%, while CoM position lowered by approximately 5%–8% during wrestling techniques in the higher FMS group. This study demonstrated the convergent validity of FMS scores with joint mobility and performance parameters in wrestling techniques. We assessed athletes’ ability to correctly perform movements using the FMS scoring system and analyzed kinematic parameters, including the displacement and velocity of the CoM, through wearable inertial sensors. Our findings indicate that higher FMS scores are associated with greater CoM velocity and the ability to maintain a low CoM position during wrestling.

## 1 Introduction

A functional movement screen (FMS) is an assessment system that identifies athletes' movement profiles and injury risks and has practical implications for determining sport-specific performance and training effectiveness (1, 2). It includes seven fundamental movement tasks; each task's completion level is scored. A score of around 14 is considered to be predictive of injury risk or low-performance levels in professional athletes (3, 4). Several studies have determined athletes' FMS scores and investigated the correlation to flexibility, mobility, and experience levels (3, 5, 6). Wrestlers with more years of sports experience achieved higher FMS scores (3). Uzer et al. (7) investigated the correlation between body posture and FMS scores in high-level wrestlers. Moreover, individual FMS tests focusing on core strength and body control were strongly associated with performance, and lower scores in the FMS were related to higher injury risk in combat sports, including karate and jiu-jitsu athletes (8, 9). Consequently, the FMS can effectively evaluate athletes’ performance levels by relating them to kinematics during sport-specific movements, utilizing wearable motion capture systems to measure technical aspects. However, none of the studies have employed the IMU measurement system to assess body kinematics during FMS tests and techniques in combat sports.

With advancements in sensor technology, inertial measurement unit (IMU) sensors have been widely utilized in both team and individual sports (10–14). Camomilla et al. (10) summarized that IMU sensors have been mostly applied in cyclic and team sports during training or simulated training sessions. Worsey et al. (11) conducted a systematic review of inertial sensors in combat sports, demonstrating that these sensors are predominantly used to assess strike quality. In the FMS study, multiple investigations have utilized IMU sensors to explore the relationship between FMS and joint mobility (15–19). Shuai (19) and Dambadarjaa (15) assessed 3D joint kinematics during various functional movements. Whiteside et al. (16) used IMU sensor-based measurement for real-time grading for FMS. They compared the FMS scores, which were assigned by a certified FMS tester, to those measured by IMU-based scoring. However, discrepancies were observed between manual and automatic FMS scoring due to each test's self-set kinematic threshold values. Spilz et al. (17) applied a deep learning approach to classify human complex movement during FMS with data collected from IMU sensors. Meanwhile, Wu et al. (18) employed a machine learning algorithm incorporating a full set of features for automatic FMS scoring, achieving higher prediction accuracy. Although they suggest that IMU sensor-based systems could potentially be applied to assessing FMS, there remains insufficient information regarding the convergent validity of FMS scoring in relation to joint mobility and performance parameters in wrestling. We propose to assess the FMS and kinematics in wrestlers using wearable IMU sensors to explore the relationship between FMS scores, ROM, and performance parameters. We hypothesized that athletes with higher FMS scores would exhibit greater joint mobility and improved wrestling performance.

## 2 Materials and methods

A cross-sectional study was conducted with two groups: a control group and a wrestling group, from April to June 2024. The testing took place in the biomechanics laboratory of the Mongolian University of Science and Technology (MUST) in Ulaanbaatar, Mongolia.

### 2.1 Participants

In this study, 20 healthy male high school students participated, comprising 10 students without a wrestling background and 10 wrestlers (Control group: age 15.3 ± 0.4 years; height 173.3 ± 3.3 cm; weight 60.5 ± 5.3 kg; Wrestler group: age 15.8 ± 1.1 years; height 170.4 ± 5.9 cm; weight 68.6 ± 8.1 kg). The control group of 10 students was chosen from MUST, drawn from the general population, while the 10 wrestlers were intentionally selected from the sports training center at the Sports Medicine and Research Center. Wrestlers were included if they were between the ages of 15 and 17, injury-free, and had a wrestling background of at least 5 years. All participants had no musculoskeletal injuries within the past year. This study was approved by the Institutional Review Board of the Mongolian University of Science and Technology and the Research Ethical Committee of the Mongolian National University of Medical Science (Ethics approval number: 2024/3-04). Before data collection, informed consent was obtained from all participants and their guardians.

### 2.2 Experimental setup

**Figure 1:**
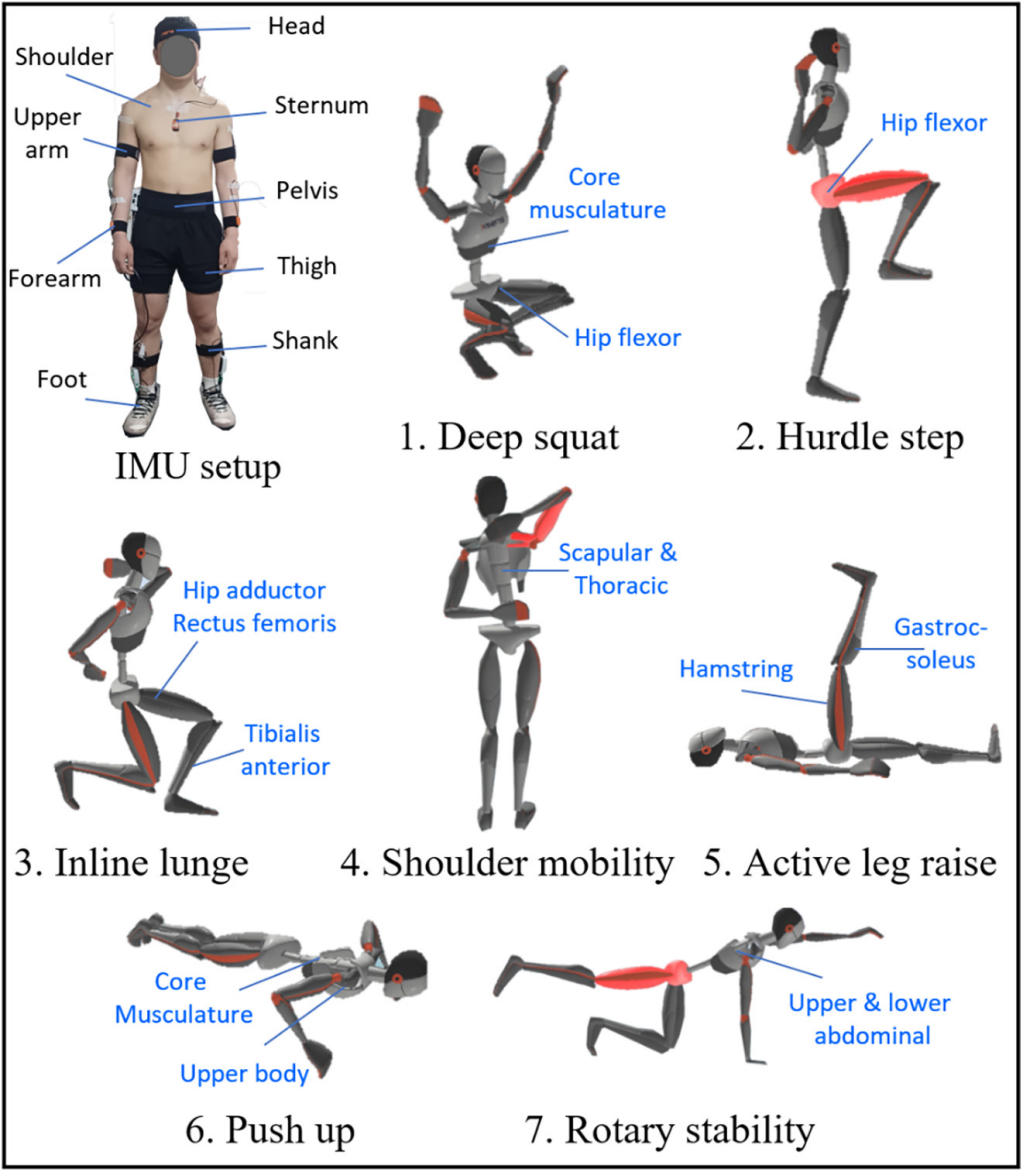
Participants wear 15 IMU sensors, and the seven movement tasks are visualized with Xsens.

The IMU sensor-based wearable motion capture system (Xsens MVN Analyze, Movella, Netherlands) was used to capture full-body joint kinematics during FMS and wrestling techniques at a sampling rate of 60 Hz with ±16 g and ±2,000°/s. Previous systematic reviews have shown that this system is one of the most commonly used commercial systems for evaluating sports performance (10). This system is composed of Xsens MVN Analyze software and hardware. The hardware includes 15 IMU sensors, a body pack, and a wireless router. The body pack connects multiple strings of 15 IMU sensors and collects their data. This data is then transmitted via a 2.4 GHz spread-spectrum wireless link to the router, which is connected to a computer (20, 21). The 15 IMU sensors were placed on the head, sternum, pelvis, left/right shoulder, upper arm and forearm, upper and lower leg, and foot (22) (Figure 1). The placement of the sensors was adjusted without interfering with participant movements (13). In the MVN Analyze software, participant height and foot length were entered to create a 23-link rigid body biomechanical model, which automatically calculated joint kinematics and the position and velocities of the CoM over time (20). Before the experiment, all subjects were asked to perform N-pose and T-pose calibration, which estimates the orientation of the sensors with respect to the corresponding segments and the proportions of the person being tracked (20). All recordings from the motion experiment, including 3D joint kinematics and the position and velocity of the CoM, were exported from the Xsens MVN Analyze software as an MVNX file, which can be opened in Microsoft Excel and MATLAB.

The control group performed only FMS tests in the experiments, while the wrestler group completed FMS tests and two different wrestling techniques. The FMS tests were deep squat, hurdle step, inline lunge, shoulder mobility, active straight leg raise, trunk stability pushup, and rotary stability according to previous studies (2, 23). Wrestling techniques were double leg attack and arm throwing techniques (24, 25). Details of wrestling techniques can be found in Section 2.4.

### 2.3 FMS score

Each test of FMS was manually scored between 0 and 3 scores while wearing the Xsens system. A higher score represents correct execution without any compensation movements. Trained physical therapists conducted manual scoring. They had previously used the FMS and worked as physical therapists for 5–10 years. Three physical therapists were involved, and the scoring was double-checked by the most experienced one. Moreover, three clearing tests were performed to assess for pain during shoulder and spinal movements. If there are any pain occurrences, a 0 score is given. Details of the scoring system can be found in (2, 23). After conducting FMS tests, scores were averaged and compared between the control and wrestler groups. Since the control and wrestler groups are unrelated, an independent *T*-test was conducted to compare their FMS scores. Differences were considered significant at *p* < 0.05. Previously, a *T*-test was used to examine differences in FMS scores between novice and experienced runners (5), healthy active males and females (26, 27). A Cohen's *d* effect size statistic was calculated for the FMS scores between control and wrestler groups. Effect sizes were interpreted as small = 0.2, medium = 0.5, and large = 0.8. Simultaneously, the three-dimensional joint kinematics were evaluated to validate the statistical differences observed in specific tests.

### 2.4 Wrestling techniques

**Figure 2:**
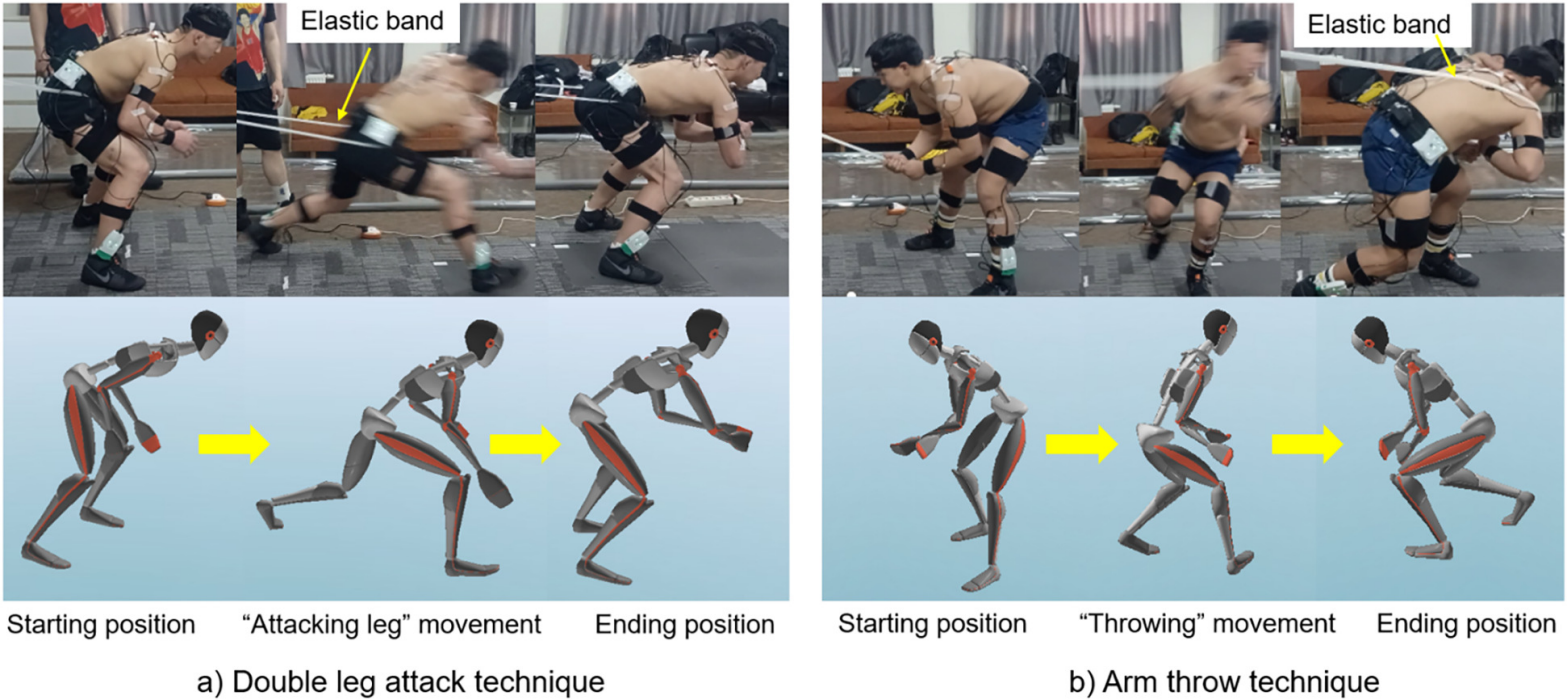
Execution of wrestling techniques with an elastic band: **(a)** double leg attack and **(b)** arm throw techniques. The arrow indicates the movement of the techniques.

Wrestlers were divided into two groups based on FMS scoring results: group A: >15 scores; group B: ≤15 scores. Groups A and B each had five wrestlers. Then, they performed two types of techniques three times with resistance elastic bands, such as double leg attack and arm throwing techniques while wearing an Xsens wearable system under the supervision of an experienced coach (Figure 2). It was reported that resistance elastic bands had been effectively used in different types of strength training environment (28).

In a double leg attack, the wrestler begins from a crouched position, steps toward the opponent with the leading foot, and ends by grabbing the opponent's knee from behind in a squat position (24). Here, wrestlers need to lower their CoM while executing moves, which helps them accelerate and enter the target position with a greater emphasis on hip abduction (Figure 2a). In arm throwing, wrestlers begin an explosive rotational movement from the lower limbs before transitioning into a crouched position. This positioning allows them to use their bodies as levers to lift and throw their opponent down. Wrestlers require enhanced movement control and a tighter grip on their opponent's arm, with a greater emphasis on their overall strength (Figure 2b) (25).

Performance parameters, such as the displacement and velocity of the CoM relative to the earth-fixed coordinate system, were calculated using Xsens MVN Analyze software. These parameters were then compared between Groups A and B. A previous study has shown that the velocity and displacement of the CoM are associated with the effectiveness of leg attacks in collegiate wrestlers (24).

## 3 Results

### 3.1 FMS scoring and selected joint kinematics

Total FMS scores were 17.2 ± 1.0 for the control group and 16.2 ± 1.6 for wrestlers, respectively (Table 1). Although no statistical differences were found overall, scores significantly differed in shoulder mobility (Control: 2.7 ± 0.6, Wrestlers: 1.9 ± 0.8) (*p* = 0.034, Cohen's *d* = 1.02) and active leg raise tests (Control: 2.3 ± 0.4, Wrestlers: 2.9 ± 0.3) (*p* = 0.004, Cohen's *d* = 1.47). Both groups achieved the highest scores on the push-up test (Control: 2.9 ± 0.3, Wrestlers: 2.9 ± 0.3), while the control group recorded the lowest score of 2.0 ± 0.4 on the rotary stability, and the wrestler group had the lowest score of 1.9 ± 0.8 on shoulder mobility.

**Table 1:** FMS scores in wrestlers and control groups.

No.	FMS tests	Control	Wrestlers	*p* value	Effect size Cohen's *d*
1	Deep squat	2.4 ± 0.5	2.2 ± 0.4	0.355	0.42
2	Hurdle step	2.4 ± 0.5	2.0 ± 0.4	0.087	0.81
3	Inline lunge	2.5 ± 0.5	2.3 ± 0.4	0.388	0.40
4	Shoulder mobility	2.7 ± 0.6	1.9 ± 0.8	0.034	1.02
5	Active leg raise	2.3 ± 0.4	2.9 ± 0.3	0.004	1.47
6	Push up	2.9 ± 0.3	2.9 ± 0.3	1	0.00
7	Rotary stability	2.0 ± 0.4	2.0 ± 0.4	1	0.00
	Total score	17.2 ± 1.0	16.2 ± 1.6	0.117	0.74

Selected joint kinematics were assessed in shoulder mobility and active leg raise tests, where the FMS scores were significantly different between groups (Table 2). Shoulder and hip range of motion (ROM) was also measured in these tests. In the shoulder mobility test, higher joint ROM was observed for groups with higher FMS scores. FMS scores were 2.7 ± 0.6 in control and 1.9 ± 0.8 in wrestlers with shoulder rotations of 91 ± 16°and 88 ± 19° (Table 2). A similar trend was observed in the active leg raise test. FMS scores were 2.3 ± 0.4 in control and 2.9 ± 0.3 in wrestlers, while hip flexion was 72 ± 5° in control and 75 ± 11° in wrestlers.

**Table 2:** ROM in shoulder mobility and active leg raise test.

Test		ROM	Control	Wrestlers
1	Shoulder mobility	Abduction	164 ± 12°	163 ± 10°
		Rotation	91 ± 16°	88 ± 19°
		Flexion	36 ± 15°	35 ± 12°
2	Active leg raise	Abduction	9 ± 6°	11 ± 7°
		Rotation	6 ± 5°	7 ± 4°
		Flexion	72 ± 5°	75 ± 11°

### 3.2 Wrestling performance parameters

**Figure 3:**
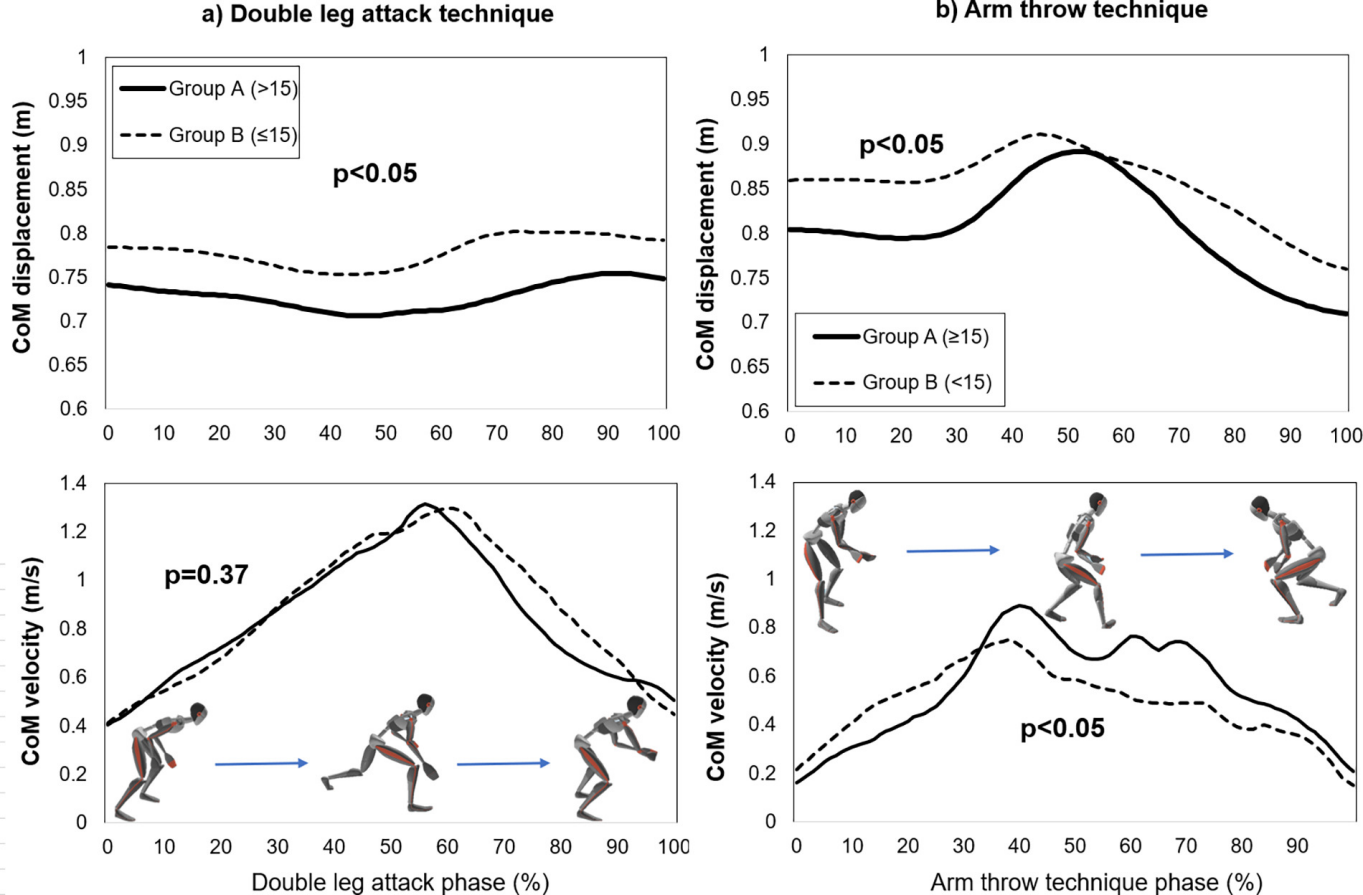
Com displacement and velocity in **(a)** double leg attack and **(b)** arm throw techniques (the lines represent the averages for each group).

The wrestlers' CoM displacement and velocity were estimated and averaged for Group A and B during double leg attack and arm throw techniques (Figure 3). During double leg attacks, Group A lowered their CoM position by about 4–5 cm more than Group B (Group A: 0.71–0.75 m, Group B: 0.75–0.80 m) (*p* < 0.05), but there was no significant difference in CoM velocity (Group A: 1.31 m/s, Group B: 1.30 m/s (*p* = 0.37) (Figure 3). Similarly, Group A lowered their CoM position by about 4 cm more than Group B (Group A: 0.71–0.89 m, Group B: 0.77–0.91 m) (*p* < 0.05). However, they showed a higher propulsive velocity of the CoM. Maximum CoM velocities were 0.89 and 0.75 m/s for Groups A and B, respectively (*p* < 0.05) (Figure 3).

## 4 Discussion

In this study, we aimed to assess FMS and kinematic parameters in wrestlers using wearable IMU sensors to explore the relationship between FMS scores, ROM, and performance parameters. First, we compared FMS scores between the control and wrestler groups. Simultaneously, joint mobility was evaluated using the wearable IMU sensors. It was found that wrestlers exhibited higher leg raise ability to disassociate the lower extremities from the trunk due to the superior development of their hamstrings and iliopsoas muscles compared to the control group. However, their shoulder mobility was lower than that of the control group; this might be related to the muscular development of the upper body. It is well known that the effectiveness of an attack is related to the velocity of movement. Therefore, wrestlers need to have a high level of muscular power in their lower limbs to execute explosive attacks and counterattacks (29). Additionally, there are sports-related characteristics that affect shoulder mobility. Wrestlers must possess greater muscle mass and power in their neck and upper body to effectively counter their opponent's offensive and defensive actions, such as waistlocks or trunk grip gut wrench techniques (30).

Those screening results were validated with sensor-based joint kinematic measurements, where the increases in shoulder and hip joint ROM were related to the higher FMS score. Similarly, Aleixo et al. (31) reported that a higher score in deep squats was characterized by larger hip, knee, and ankle joint flexion. Moreover, wrestlers with higher FMS scores demonstrate greater CoM velocity and tend to maintain a low-level CoM position during wrestling techniques. Those who scored 15 or higher had an 18% faster CoM velocity and a 9% lower CoM position during arm throwing. Lowering the body while executing the wrestling techniques quickly gives more chances to take down the opponents by producing enough mechanical force over counterparts and disrupting the opponent's balance. It was previously observed that athletes with better FMS scores improved performance by up to 2.9% (32). Also, deep squat scores impacted the performance of track and field athletes (32). Davies et al. (33) concluded that children and youth who scored high on FMS tended to perform better. But, there was not much difference in CoM velocity during a double-leg attack. It was similar that CoM propulsive velocity was not different between elite and non-elite groups during leg attacks (24). It should be noted that the duration of movement is not necessarily necessary during the double attack, but it was crucial in arm throwing.

A study has several limitations. The sample size was small and limited to high school wrestlers, lacking diversity and representativeness. Additionally, the control group has no wrestling experience, which may introduce bias in the results. It is also important to note that this study does not delve into the causal relationship between FMS scores and athletic performance. Future directions involve examining the correlation between FMS scores and full-body kinematics, along with other indicators of physical performance among a larger number of professional athletes using comprehensive statistical analysis.

In conclusion, we demonstrated the convergent validity of FMS scores in relation to joint mobility and wrestling performance parameters. We evaluated athletes' ability to perform movements correctly using the FMS scoring system and analyzed kinematic parameters, including the displacement and velocity of the CoM, using wearable inertial sensors. Our findings indicate that higher FMS scores are associated with greater CoM velocity and the ability to maintain a low-level CoM position during wrestling. Therefore, the FMS can effectively assess athletes' movement profiles and performance levels. This highlights the FMS as a valuable tool for evaluating athlete performance and its potential applications for training correction and injury prevention in wrestlers.

## Data Availability

The original contributions presented in the study are included in the article/Supplementary Material, further inquiries can be directed to the corresponding author.
